# Upregulation of Relaxin after Experimental Subarachnoid Hemorrhage in Rabbits

**DOI:** 10.1155/2014/836397

**Published:** 2014-07-16

**Authors:** Yuichiro Kikkawa, Satoshi Matsuo, Ryota Kurogi, Akira Nakamizo, Masahiro Mizoguchi, Tomio Sasaki

**Affiliations:** Department of Neurosurgery, Graduate School of Medical Sciences, Kyushu University, 3-1-1 Maidashi, Higashi-ku, Fukuoka, Fukuoka 812-8582, Japan

## Abstract

*Background*. Although relaxin causes vasodilatation in systemic arteries, little is known about its role in cerebral arteries. We investigated the expression and role of relaxin in basilar arteries after subarachnoid hemorrhage (SAH) in rabbits. *Methods*. Microarray analysis with rabbit basilar artery RNA was performed. Messenger RNA expression of relaxin-1 and relaxin/insulin-like family peptide receptor 1 (RXFP1) was investigated with quantitative RT-PCR. RXFP1 expression in the basilar artery was investigated with immunohistochemistry. Relaxin concentrations in cerebrospinal fluid (CSF) and serum were investigated with an enzyme-linked immunosorbent assay. Using human brain vascular smooth muscle cells (HBVSMC) preincubated with relaxin, myosin light chain phosphorylation (MLC) was investigated with immunoblotting after endothelin-1 stimulation. *Results*. After SAH, RXFP1 mRNA and protein were significantly downregulated on day 3, whereas relaxin-1 mRNA was significantly upregulated on day 7. The relaxin concentration in CSF was significantly elevated on days 5 and 7. Pretreatment with relaxin reduced sustained MLC phosphorylation induced by endothelin-1 in HBVSMC. *Conclusion*. Upregulation of relaxin and downregulation of RXFP1 after SAH may participate in development of cerebral vasospasm. Downregulation of RXFP1 may induce a functional decrease in relaxin activity during vasospasm. Understanding the role of relaxin may provide further insight into the mechanisms of cerebral vasospasm.

## 1. Introduction

Cerebral vasospasm is one of the most important cerebrovascular events following subarachnoid hemorrhage (SAH) and is characterized by delayed and prolonged contraction of cerebral arteries that may cause cerebral ischemia and lead to death or neurological deficits in patients with SAH [1]. Therefore, the prevention as well as treatment of vasospasm is important in the management of SAH patients. Although increased production of spasmogens and increased vascular responsiveness can be attributed to cerebral vasospasm, the mechanism of cerebral vasospasm remains elusive, and thus effective therapeutic strategies are not available. Recent randomized clinical trials have shown that currently available antivasospastic drugs are not sufficient to improve outcome [2]. Therefore, further research efforts are needed to clarify the mechanism of vasospasm and find new therapeutic targets.

Relaxin is a small peptide hormone (6 kDa) that is primarily produced by the corpus luteum, decidua, and placenta during pregnancy [3]. Three relaxin genes have been identified in humans and are designated as relaxin-1 (*RLN1*), relaxin-2 (*RLN2*), and relaxin-3 (*RLN3*). Human relaxin-2 is the only form of circulating relaxin that is substantially increased during pregnancy [4]. Human relaxin-2 is functionally equivalent to relaxin-1 in all other mammals [5]. Recently,* RLN* mRNA expression has also been detected in nonreproductive tissues including arteries, heart, kidney, liver, and lung [6–8].

Four relaxin receptor genes have been identified. They are relaxin/insulin-like family peptide receptors and are named RXFP1 (*RXFP1*), RXFP2 (*RXFP2*), RXFP3 (*RXFP3*), and RXFP4 (*RXFP4*) [8]. Circulating relaxin (relaxin-2 in humans and relaxin-1 in other mammals) binds to RXFP1 (previously known as leucine-rich repeat-containing G-protein-coupled receptor 7: LGR 7) with high affinity [7, 9, 10].* RXFP1* mRNA and protein are expressed in a wide range of reproductive tissues including ovary, uterus, placenta, mammary gland, prostate, and testis [8]. The receptor is also expressed in nonreproductive tissues including heart, kidney, lung, liver, and vasculature [8]. Beyond a role in the reproductive system during pregnancy, a growing body of literature suggests that relaxin has extensive cardiovascular effects such as promoting vasodilation and angiogenesis and protecting against fibrosis and inflammation in systemic and renal circulation [11, 12].

Several recent studies have reported that relaxin and RXFP1 (LGR7) are expressed in the local arteries of mice and rats [13, 14]. These molecules are localized in the local arterial wall and seem to contribute to increased arterial compliance and reduced myogenic reactivity, and they may mediate blood flow to tissues [13, 14].

Based on the potent cardiovascular effects of relaxin, we hypothesize that relaxin dilates the cerebral arteries and plays a role in mediating cerebral blood flow. To date, however, no study has explored the expression and role of relaxin and its receptor, RXFP1, in the cerebral arteries after SAH. Therefore, the purpose of the present study was to investigate the time course of* RLN* and* RXFP1* expression in the cerebral arteries after SAH and to clarify the role of relaxin during vasospasm.

## 2. Materials and Methods

### 2.1. Preparation of the Rabbit SAH Model

This study was performed in accordance with the guidelines for proper conduct of animal experiments published by the Science Council of Japan. The study protocol was approved by the Animal Care and Use Committee, Kyushu University (Permit number A24-103-0). Adult male Japanese white rabbits (2.5 to 3.0 kg) were anesthetized with an intramuscular injection of ketamine (40 mg/kg body weight) and given an intravenous injection of sodium pentobarbital (20 mg/kg body weight). On day 0, 0.5 mL cerebrospinal fluid (CSF) was aspirated percutaneously from the cisterna magna using a 23-gauge butterfly needle, and then 2.5 mL nonheparinized autologous arterial blood that was obtained from the central ear artery was injected into the cisterna magna over 1 minute. The animal was kept in a prone position with the head tilted down at 30° for 30 minutes. During this procedure, no blood clot formation was observed in the syringe. On day 2, a similar second injection of autologous blood was performed. In this study, rabbits that did not undergo any surgical procedures including puncturing of skin or dura mater with a needle were used as control model (day 0). One of the reasons why nonmanipulated rabbit was used as the control model is to curb the number of rabbits used as possible from the point of view of animal ethics.

### 2.2. Harvest of Rabbit Basilar Artery

On days 0, 3, 5, and 7 after the first hemorrhage, the rabbits were heparinized (400 U/kg body weight), euthanized by intravenous injection of an overdose of sodium pentobarbital (120 mg/kg body weight), and exsanguinated from the common carotid artery. Exposure of the brain revealed clot formation over the surface of the pons and the basilar artery in the SAH animals. Immediately after removing the whole brain* en bloc*, the subarachnoid membrane was carefully dissected, and the clot was gently removed under a binocular microscope (Leica EZ4D, Leica Microsystems, Wetzler, Germany) with microscissors and microforceps so as not to touch the basilar artery. The distal half of the surface of the basilar artery in particular was covered with a thick clot in all rabbits with SAH. To estimate vasospasm, the external diameter of the basilar artery was measured at a location that was one-third the length from the distal end of the basilar artery. The ventral surface of the whole brain was photographed with a digital camera (CX3, Richo, Tokyo, Japan), and the external diameter of the basilar artery was analyzed with ImageJ (National Institutes of Health, Bethesda, MD, USA). The entire length of the basilar artery was then immediately excised from the brain and dissected free from surrounding tissues with microscissors and microforceps. Intraluminal blood was gently hand-flushed out with normal physiological salt solution (123 mmol/L NaCl, 4.7 mmol/L KCl, 1.25 mmol/L CaCl_2_, 1.2 mmol/L MgCl_2_, 1.2 mmol/L KH_2_ PO_4_, 15.5 mmol/L NaHCO_3_, and 11.5 mmol/L d-glucose) using a tuberculin syringe, and then the basilar artery was frozen in liquid nitrogen and stored at −80°C until use.

### 2.3. Total RNA Isolation from the Rabbit Basilar Artery

Total RNA was extracted from the basilar artery using the TRIZOL Reagent (Invitrogen, Carlsbad, CA), according to the manufacturer's protocols. The quality of total RNA was evaluated with a spectrophotometer (Nano-Drop2000c; Thermoscientific, Wilmington, DE) and gel electrophoresis (Experion; Bio-Rad, Hercules, CA, USA). RNA samples with an A260/280 ratio higher than 1.8 were used for quantitative real-time polymerase chain reaction (qRT-PCR) analysis. In addition to evaluation with a spectrophotometer, RNA samples with an RNA Quality Index higher than 9.0 were used for gene expression microarray analysis.

### 2.4. Gene Expression Microarray Analysis of the Rabbit Basilar Artery

Total RNAs extracted from rabbit basilar arteries on days 0, 3, 5, and 7 after the first hemorrhage (*n* = 3 each) were used for the microarray analysis. From 50 ng total RNA, cRNA was amplified, labeled, and hybridized to a rabbit gene expression microarray (Agilent Technologies, Santa Clara, CA, USA) using the Low Input Quick Amp one-color Labeling kit (Agilent Technologies) according to the manufacturer's instructions. All hybridized microarray slides were scanned with an Agilent Microarray scanner G2505B (Agilent Technologies). Relative hybridization intensities and background hybridization values were calculated using Agilent Feature Extraction Software (9.5.1.1) (Agilent Technologies). According to the manufacturer's instructions, raw signal intensities and flags for each probe were calculated from hybridization intensities and spot information. The raw signal intensities of the samples were log_2_ transformed and normalized using a quantile algorithm with the “preprocessCore” library package of Bioconductor software [15, 16]. Then, we identified differentially expressed genes in the SAH model using the linear models for microarray analysis (limma) package of Bioconductor software [16, 17]. Genes in SAH samples with a limma value of *P* < 0.05 and an absolute limma log_2_ fold change (|log_2_ fold change|) higher than 1.0 compared to the control samples were defined as differentially expressed genes in this study. Microarray data are available from the Gene Expression Omnibus (GEO, http://www.ncbi.nlm.nih.gov/geo/) with the accession number GSE44910.

### 2.5. Quantitative RT-PCR Analysis of mRNA Expression of* RLN1* and* RXFP1* in the Rabbit Basilar Artery

Total RNAs extracted from rabbit basilar arteries on days 0, 3, 5, and 7 after the first hemorrhage were used for RT-PCR (*n* = 5 each). Complementary DNA (cDNA) was synthesized at 42°C for 30 minutes using 200 ng RNA template in a 20 *μ*L reaction mixture containing high capacity RNA-to-cDNA master mix (Applied Biosystems, Foster City, CA, USA) with an ABI 2720 thermal cycler (Applied Biosystems). The cDNA was stored at −80°C until use in qRT-PCR. qRT-PCR was performed in triplicate in a 20 *μ*L reaction mixture containing TaqMan Fast Universal PCR master mix (Applied Biosystems), 10 ng cDNA, and components of the TaqMan gene expression assay kit (rabbit* RLN1*: Oc03398001_m1, Applied Biosystems) or Custom TaqMan gene expression assay kit (rabbit* RXFP1*: forward primer 5′-GCATTCTCCAGAGAGTGTTTGTCT-3′, reverse primer 5′-GGCGCATGCAGATGACAAAA-3′, TaqMan probe 5′-ACTGCGGAGACCACC-3′, Applied Biosystems) using an ABI 7500 Fast Real-Time PCR system thermal cycler (Applied Biosystems). Glyceraldehyde 3-phosphate dehydrogenase (rabbit* GAPDH*: Oc03823402_g1) was amplified as an endogenous control. The PCR protocol was composed of initial denaturation at 95°C for 20 seconds, followed by 40 amplification cycles of 95°C for 3 seconds and 60°C for 30 seconds. The data were analyzed with the comparative cycle method. The relative amount (*X*_0_/*R*_0_) of target gene mRNA was calculated with the Ct value for the target gene mRNA (CtX) and the Ct value for GAPDH mRNA (CtR) in the same sample using the formula *X*_0_/*R*_0_ = 2^CtX−CtR^, where *X*_0_ is the original amount of target gene mRNA and *R*_0_ is the original amount of GAPDH. The level of target gene expression on day 0 was assigned a value of 100%. The data were expressed as the mean values ± SEM.

### 2.6. Enzyme-Linked Immunosorbent Assay (ELISA) Analysis of Relaxin in Rabbit Cerebrospinal Fluid (CSF) and Serum

Rabbit CSF (*n* = 6) and serum (*n* = 15) samples were collected on days 0, 3, 5, and 7. After rabbits were anesthetized and positioned as described above, CSF (0.5 mL) and arterial blood (2 mL) were collected from the cisterna magna and central auricular artery, respectively. CSF samples were centrifuged at 10,000 rpm for 5 minutes at 4°C to remove red blood cells, nonactivated platelets, and other cellular debris. The supernatant was then frozen at −80°C for later analysis. Rabbit arterial blood (2 mL) was collected from the rabbit ear artery and transferred into anticoagulant-free vacuum tubes (Venoject VP-AS109 K50, Terumo Corporation, Tokyo, Japan). After centrifugation at 1,500 ×g for 15 minutes at 4°C, the serum was pipetted into tubes and frozen at −80°C for later analysis. For detection and quantification of the relaxin concentration in rabbit CSF and serum, a commercially available Relaxin ELISA kit (USCN Life Science, Wuhan, China) was used according to the manufacturer's instructions. Standards and all samples were measured in duplicate.

### 2.7. Perfusion Fixation of the Rabbit Basilar Artery

On days 0, 3, 5, and 7 (*n* = 3 each) after the first hemorrhage, rabbits were anesthetized as described above. A thoracotomy was performed, the left ventricle was cannulated with an 18 G catheter (Surflo IV Catheter, Terumo Corporation), the right atrium was opened widely, and the descending thoracic aorta was clamped. Perfusion was begun with 500 mL heparinized saline (10 U/mL), followed by 500 mL 4.0% paraformaldehyde through a cannula in the left ventricle with a perfusion pressure of 100 cmH_2_O. The brain including the basilar artery was removed and stored in 4.0% paraformaldehyde solution at 4°C overnight. For histological examination, the brain including the basilar artery was embedded in paraffin and cut into 6 *μ*m sections.

### 2.8. Immunohistochemical Staining Analysis of* RXFP1* Expression in the Rabbit Basilar Artery

The sections were deparaffinized, and endogenous peroxidase activity was quenched by incubation in 3% H_2_O_2_ in methanol for 30 minutes at room temperature. Heat-induced epitope retrieval was performed at 120°C for 20 minutes in 0.01 M citrate buffer. The sections were then incubated with blocking serum (Vectastain Elite ABC kit, Vector Laboratories, Burlingame, CA) followed by incubation with monoclonal anti-mouse LGR7 (RXFP1) antibody (1 : 100, Sigma-Aldrich, St. Louis, MO, USA) at 4°C overnight. Sections were incubated with biotinylated horse anti-mouse antibodies and then incubated with avidin-horseradish peroxidase (HRP) conjugate (Vector Laboratories, Burlingame, CA, USA) according to the manufacturer's recommendations for the Vecstain Elite ABC kit. The staining procedure was performed using a Vectastain Elite ABC kit (Vector Laboratories) and 3,3′-diaminobenzidine (Dojindo Laboratories, Kumamoto, Japan) according to the manufacturer's instructions. The sections were then counterstained with hematoxylin and observed under a microscope (BZ-8000, Keyence, Osaka, Japan). Quantitative analysis was performed by modification of previously described methods [18]. Immunoreactivity was quantitatively analyzed by placing a 25 × 25 *μ*m square over the most intensely stained area in the tunica media, and densitometric analysis was performed with image analysis software (BZ-Analyzer, Keyence).

### 2.9. Cell Culture, Treatment, and Protein Extraction

Human brain vascular smooth muscle cells (HBVSMC) were obtained from ScienCell Research Laboratories (Carlsbad, CA, USA) and cultured in smooth muscle cell medium, which consists of 500 mL basal medium, 10 mL fetal bovine serum, 5 mL smooth muscle cell growth supplement, and 5 mL penicillin/streptomycin solution. Cell cultures were maintained at 37°C in a humidified atmosphere with 5% CO_2_. Cells from passages 5 to 10 were used for the study.

For experiments, cells were seeded at 3–5 × 10^5^ cells/6 cm dish and grown until 90% confluent. Then, cells were incubated in serum-free smooth muscle cell medium (ScienCell Research Laboratories) in the presence or absence of 10 nmol/L human recombinant relaxin-2 (H2-relaxin, Peprotech, Rocky Hill, NJ, USA) for 24 hours. After stimulation with 100 nmol/L endothelin-1 (Peptide Institute, Osaka, Japan) for 0, 5, 10, and 30 minutes with or without relaxin treatment, smooth muscle cell proteins were extracted as follows.

At the indicated time points, cells were rinsed three times with ice-cold phosphate-buffered saline (PBS: 136.9 mmol/L NaCl, 2.7 mmol/L KCl, 8.1 mmol/L Na_2_HPO_4_ 12H_2_O, and 1.47 mmol/L KH_2_PO_4_) and lysed with ice-cold cell lysis buffer (50 mmol/L HEPES, pH 7.4, 150 mmol/L NaCl, 0.5% (v/v) Nonidet P-40, and 5 *μ*mol/L microcystin-LR). Immediately after scraping the cells off the dishes, cell lysates were snap-frozen in liquid nitrogen and thawed at room temperature. After centrifugation for 15 minutes at 12,000 rpm at 4°C, the supernatants were collected. The protein concentrations of the supernatants were measured using the Bradford method (Thermo Fisher Scientific, Waltham, MA, USA). The samples were diluted to a final concentration of 1 mg/mL with the cell lysis buffer and 4 × LDS NuPAGE sample buffer (Invitrogen) and stored at −80°C until use.

### 2.10. Analysis of Myosin Light Chain (MLC) Phosphorylation with Phos-Tag Sodium Dodecyl Sulfate-Polyacrylamide Gel Electrophoresis (SDS-PAGE)

Phosphorylation of MLC was analyzed using a new method based on Phos-tag technology. Phos-tag is a compound that specifically binds to phosphate groups. Therefore, SDS-PAGE containing polyacrylamide-bound Mn^2+^ Phos-tag (Phos-tag SDS-PAGE) causes a mobility shift, depending on the degree of phosphorylation [19].

Before electrophoresis, the samples were heated at 100°C for 5 minutes and equilibrated to room temperature. The 2 *μ*g protein samples were separated on 12.5% (w/v) polyacrylamide gels containing 30 *μ*M Phos-tag Acrylamide (NARD Institute, Hyogo, Japan) for SDS-PAGE and transferred to polyvinylidene difluoride membranes (Bio-Rad). Electrophoresis was performed in 0.1% (w/v) SDS, 25 mmol/L Tris-hydroxymethyl aminomethane, and 192 mmol/L glycine at 12 mA constant current/8 cm × 5 cm × 0.75 mm gel for 155 minutes. After electrophoresis, the gel was soaked for 30 minutes in transfer buffer (25 mmol/L Tris, 192 mmol/L glycine, and 10% (v/v) methanol) containing 2 mmol/L EDTA to remove the Mn^2+^ and then in transfer buffer without EDTA for 15 minutes. Proteins were then transferred to polyvinylidene difluoride membranes (0.2 *μ*m pore size; Bio-Rad) in transfer buffer for 2 hours at room temperature. The membranes were then washed in PBS for 5 minutes and treated with 0.5% (w/v) formaldehyde in PBS for 45 minutes. After a brief wash in PBS, the membrane was blocked with 5% (w/v) skimmed milk in T-TBS overnight at 4°C. All forms of 20 kDa MLCs were detected on the immunoblot using a rabbit polyclonal anti-MLC antibody (1 : 500; Santa Cruz Biotechnology, Santa Cruz, CA, USA) and horseradish peroxidase-conjugated goat anti-rabbit IgG antibody (1 : 1000; Sigma) diluted in immunoreaction enhancer solution (Can Get Signal; Toyobo, Osaka, Japan). The immune complex was detected using enhanced chemiluminescence (ECL plus kit; Amersham, Buckinghamshire, UK). The light emission was detected and analyzed with VersaDoc 5000 and the computer program Quantity One (Bio-Rad). The percent of phosphorylated MLC of the total MLC (sum of unphosphorylated and phosphorylated forms) was calculated to indicate the extent of MLC phosphorylation.

### 2.11. Statistical Analysis

The data are expressed as the mean value ± SEM of the indicated experimental number. One basilar arterial preparation obtained from one animal was used for each experiment, and therefore the number of experiments (*n* value) equals the number of rabbits. An analysis of variance followed by Dunnett's post hoc test was used to determine statistically significant differences in a multiple comparison with the control model. A value of *P* < 0.05 was considered statistically significant. All analyses were performed using GraphPad PRISM software version 5.0 (GraphPad software, San Diego, CA, USA).

## 3. Results

### 3.1. Assessment of Cerebral Vasospasm following SAH

**Figure 1:**
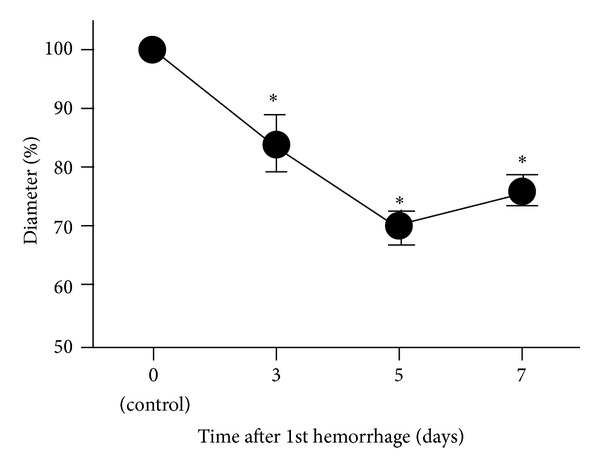
Time course of narrowing of the rabbit basilar artery after subarachnoid hemorrhage (SAH). Time course of changes in the external diameter of rabbit basilar arteries after SAH. Data are the means ± SEM (*n* = 3, each time point). The external diameter on day 0 was considered 100%. *P < 0.05 versus day 0 (control).

The external diameter of the basilar artery was 0.87 ± 0.048 mm in the controls (day 0, before SAH induction). On day 3, the basilar artery was significantly narrowed to 84.0 ± 4.7% of the control (*P* < 0.05). The basilar arterial narrowing peaked on day 5 (70.1 ± 2.8% of the control, *P* < 0.05) and then persisted to day 7 (76.1 ± 2.6% of the control, *P* < 0.05) (Figure 1).

### 3.2. mRNA Expression of* RLN1* and* RXFP1* in the Rabbit Basilar Artery after SAH and Microarray Analysis of the Rabbit Basilar Artery

**Figure 2:**
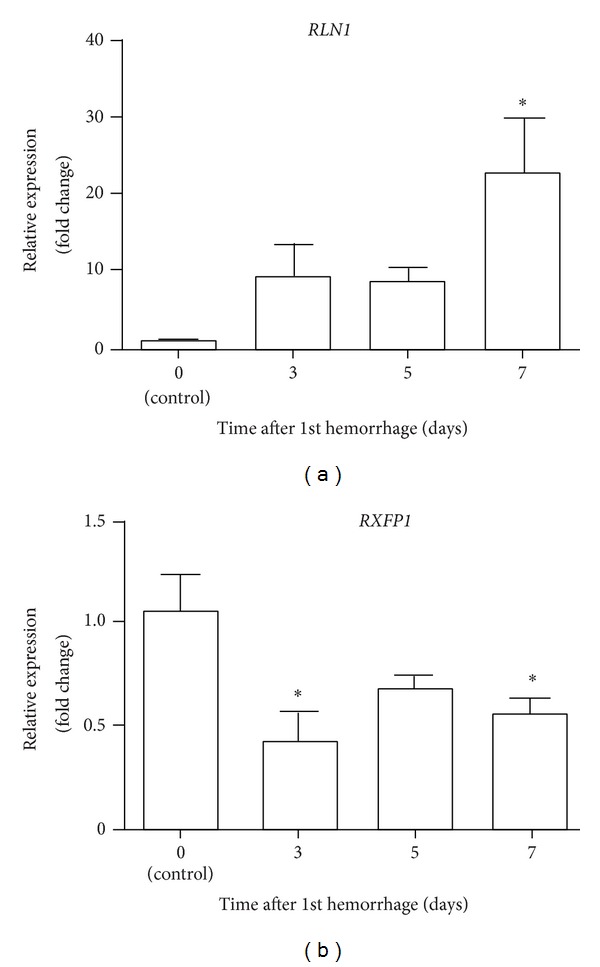
Time course of changes in mRNA expression of* RLN1* and* RXFP1* in the rabbit basilar artery after subarachnoid hemorrhage (SAH). Quantitative real-time polymerase chain reaction analysis of* RLN1* (a) and* RXFP1* (b) mRNA expression in the basilar artery after SAH. Data are the means ± SEM (*n* = 5). The level of mRNA expression on day 0 was considered 1.0. *P < 0.05 versus day 0 (control).

The three most significantly upregulated genes in the rabbit basilar artery after SAH on days 3, 5, and 7 compared to day 0 were identified from our previous microarray data (GEO accession number GSE44910) and are listed in Table 1 [20]. Among all investigated differentially expressed genes,* RLN1* was the most upregulated gene on days 5 and 7. The log_2_ fold changes for* RLN1* were 2.1, 4.7, and 4.8 on days 3, 5, and 7, respectively. We focused on* RLN1* for further investigation because the* RLN1* mRNA was markedly upregulated on days 5 and 7 when delayed cerebral vasospasm became more severe. To confirm the* RLN1* mRNA expression seen in the microarray, qRT-PCR was performed and showed that the expression of* RLN1* mRNA was significantly upregulated on day 7 compared to day 0 (Figure 2(a)). Next, we investigated the expression of* RXFP1*, which is a specific receptor for* RLN1* that is not present on the microarray chip we used. Quantitative RT-PCR revealed that* RXFP1* mRNA was significantly downregulated on days 3 and 7 (Figure 2(b)).* RLN1* mRNA was gradually upregulated after SAH, whereas* RXFP1* mRNA was persistently downregulated immediately after SAH.

### 3.3. Time Course of Changes in the Relaxin Concentration in CSF and Serum in Rabbits after SAH

**Figure 3:**
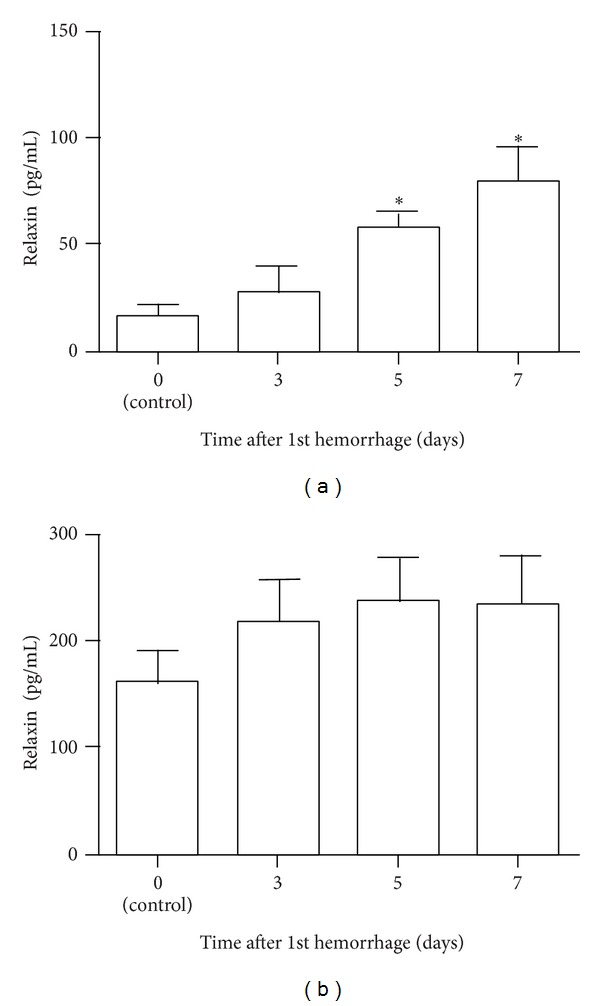
Time course of changes in the concentration of relaxin in cerebrospinal fluid (CSF) and serum in rabbits after subarachnoid hemorrhage (SAH). Enzyme-linked immunosorbent assay analysis of the relaxin concentration in the CSF (a) and serum (b) of rabbits after SAH. Data are the means ± SEM (CSF *n* = 6, serum *n* = 15). *P < 0.05 versus day 0 (control).

We performed ELISA to measure the relaxin concentration in the rabbit CSF and serum after SAH. Before SAH induction, the relaxin concentration in CSF was 16.5 ± 5.6 pg/mL. After SAH, the relaxin concentration in CSF increased gradually and peaked on day 7. The relaxin concentrations in CSF on days 3, 5, and 7 were 27.9 ± 11.8 pg/mL, 58.3 ± 6.9 pg/mL, and 79.9 ± 15.9 pg/mL, respectively, with significant elevation on days 5 and 7 (Figure 3(a)). The time course of elevation of the relaxin concentration in CSF was consistent with the upregulation of RLN1 mRNA in the basilar artery.

The relaxin concentration in serum was 161.5 ± 29.1 pg/mL before induction of SAH, and on days 3, 5, and 7, the concentrations were 219.5 ± 38.7 pg/mL, 237.5 ± 40.6 pg/mL, and 233.9 ± 46.7 pg/mL, respectively. No significant difference was found between control and SAH samples at any time (Figure 3(b)).

### 3.4. Localization and Expression of* RXFP1* in the Rabbit Basilar Artery

**Figure 4:**
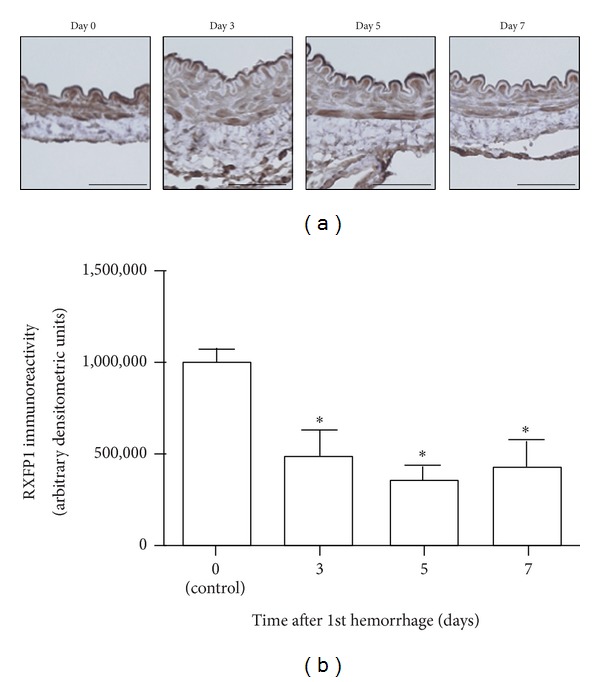
Time course of changes in RXFP1 expression in the rabbit basilar artery after subarachnoid hemorrhage (SAH). (a) Representative images of immunohistochemical staining for RXFP1 on cross sections of rabbit basilar arteries after SAH (scale bars, 50 *μ*m). (b) A summary of densitometric analysis of immunohistochemical staining for RXFP1. Data are the means ± SEM (*n* = 3 per group). *P < 0.05 versus day 0 (control).

To assess the localization of RXFP1 expression in the rabbit basilar artery, immunohistochemical analysis of RXFP1 was performed. RXFP1-positive cells were observed in all layers of the rabbit basilar artery, especially in the media tunica of both control (day 0) and SAH (days 3, 5, and 7) samples (Figure 4(a)). Immunoreactivity for RXFP1 was seen on day 0, indicating that RXFP1 was constitutively expressed in normal basilar arteries of rabbits.

Densitometry demonstrated that RXFP1 immunoreactivity on days 3, 5, and 7 was significantly decreased compared to day 0. Arbitrary densitometric units for each day were as follows: 1,008,818.6 ± 60,874.8 on day 0, 489,367.1 ± 139,762.8 on day 3 (*P* < 0.05), 359,591.3 ± 76,946.9 on day 5 (*P* < 0.05), and 427,643.9 ± 147,666.8 on day 7 (*P* < 0.05) (Figure 4(b)). The time course of RXFP1 immunoreactivity was consistent with that of* RXFP1* mRNA expression.

### 3.5. Phosphorylation of MLCs during Endothelin-1 Stimulation of Cultured HBVSMC

Phos-tag SDS-PAGE followed by immunoblot detection with anti-MLC antibody yielded three bands, as previously reported [21]. The upper, middle, and lower bands were di-, mono-, and nonphosphorylated forms of MLC, respectively. HBVSMC contained 71.0 ± 3.5% mono- and di-phosphorylated forms of MLC before endothelin-1 stimulation (*n* = 3).

**Figure 5:**
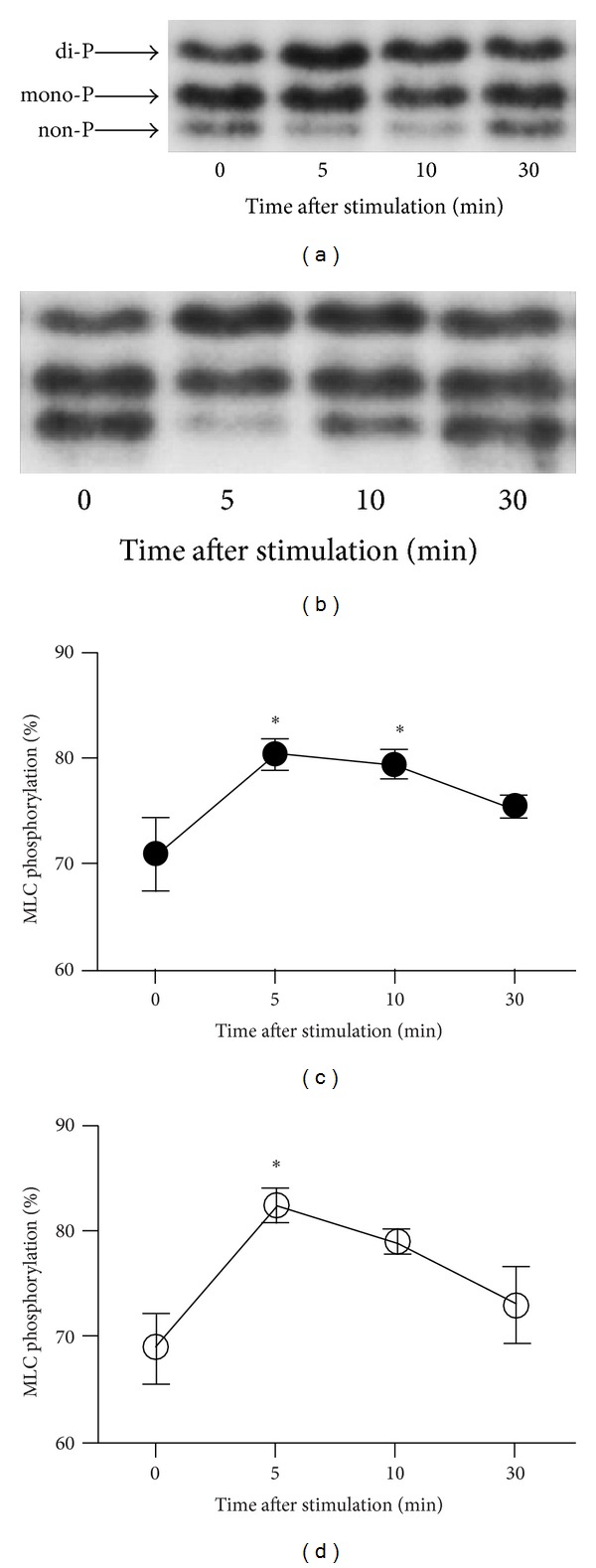
Time course of changes in myosin light chain (MLC) phosphorylation induced by endothelin-1 in human brain vascular smooth muscle cells (HBVSMC). Representative immunoblot analysis of MLC phosphorylation in HBVSMC preincubated without (a) and with (b) human recombinant relaxin-2 (H2 relaxin). The upper, middle, and lower bands detected with anti-MLC antibody represented di-, mono-, and nonphosphorylated forms of MLC, respectively. Summary of the level of MLC phosphorylation (percent of total MLC) induced by 100 nmol/L endothelin-1 at four time points: just before relaxin stimulation (0 minutes) and 5, 10, and 30 minutes after relaxin stimulation ((c) preincubated without H2 relaxin, (d) preincubated with H2 relaxin). The data are the means ± SEM (*n* = 3). *P < 0.05 versus 0 minutes.

HBVSMC preincubated with relaxin contained 68.9 ± 3.2% phosphorylated MLC before endothelin-1 stimulation (*n* = 3). No significant difference in the resting level of MLC phosphorylation (the sum of di- and mono-phosphorylation) was found between HBVSMC preincubated with or without relaxin. Endothelin-1 (100 nmol/L) significantly increased the level of MLC phosphorylation in HBVSMC preincubated with (82.1 ± 1.6%, *P* < 0.05) and without (80.4 ± 1.4%, *P* < 0.05) relaxin 5 minutes after stimulation (*n* = 3). Ten minutes after endothelin-1 stimulation, the level of MLC phosphorylation was still significantly elevated in HBVSMC not preincubated with relaxin (79.5 ± 1.3%, *P* < 0.05), whereas it declined in HBVSMC preincubated with relaxin (78.7 ± 1.1%, *P* > 0.05) (*n* = 3). Then, the level of MLC phosphorylation declined in HBVSMC preincubated both with (72.9 ± 3.7%, *P* > 0.05) and without (75.4 ± 1.0%, *P* > 0.05) relaxin 30 minutes after endothelin-1 stimulation (*n* = 3) (Figure 5).

## 4. Discussion 

In this study, we demonstrated that relaxin and RXFP1 are expressed in the rabbit basilar artery and that their expression was altered after SAH.* RLN1* mRNA was gradually upregulated after SAH, whereas* RXFP1* mRNA and protein were downregulated just after SAH. Moreover, the relaxin concentration in CSF was elevated after SAH, and the time course of elevation of relaxin in CSF was consistent with the progression of cerebral vasospasm. We also demonstrated that pretreatment with human recombinant relaxin-2 reduced the sustained MLC phosphorylation that was caused by endothelin-1 stimulation in HBVSMC, suggesting that relaxin inhibits the endothelin-1-induced sustained contraction in the cerebral arteries. These data suggest that upregulation of relaxin-1 and downregulation of RXFP1 play an important role in the development of cerebral vasospasm.

Relaxin affects several organs and exerts multiple effects including vasodilatation, antifibrosis, and anti-inflammation, and this peptide maintains organ blood flow via RXFP1 [8, 14, 22]. Several studies have demonstrated that relaxin dilates arteries [23, 24]. Our in vitro data demonstrated that relaxin reduced the sustained MLC phosphorylation after endothelin-1 stimulation. Therefore, relaxin has the potential to dilate cerebral arteries. Immunohistochemistry and qRT-PCR demonstrated that downregulation of* RXFP1* occurred 3 days after SAH and persisted to day 7 in the rabbit basilar artery. A previous study in rats reported that downregulation of RXFP1 expression in the uterus reduces the smooth muscle relaxation activity of relaxin [25]. Therefore, the vasodilatory effect of relaxin on cerebral arteries may be reduced in the rabbit basilar artery after SAH. A functional decrease in relaxin activity caused by RXFP1 downregulation seems to be involved in the development of cerebral vasospasm. Because relaxin exerts multiple effects on several organs, further studies are needed to elucidate the effects of relaxin on cerebral arteries.

The mechanism of regulation of RXFP1 expression in the artery remains unknown. Expression of* RLN3* mRNA may contribute to the mechanism of* RXFP1* downregulation in rat heart [6]. In this study,* RLN3* mRNA was not detected in the rabbit basilar artery at any time point investigated (data not shown). Moore et al. proposed that *β*-adrenergic receptor activation, especially *β*1-adrenergic receptors, suppresses the expression of* RXFP1* mRNA in rat heart [26]. Adrenaline is a representative spasmogen [1] and is increased in plasma and CSF after SAH [27]. The elevated expression of adrenaline may participate in the mechanism of RXFP1 downregulation in the basilar arteries after SAH.

Luteral relaxin expression is regulated by luteinizing hormone, chorionic gonadotrophin, and basic fibroblast growth factor [28]. However, the mechanism of regulation of the expression of arterial-derived relaxin remains elusive. Our data demonstrated that downregulation of RXFP1 was followed by upregulation of relaxin-1. One explanation for this finding may be that a receptor-ligand feedback mechanism exists to regulate the expression of the ligand. However, further research is needed to identify the mechanism of regulation of expression of relaxin-1 and RXFP1 in the cerebral arteries after SAH.

The concentration of relaxin in CSF was significantly elevated after SAH, and the time course was consistent with that of* RLN1* mRNA expression in the rabbit basilar artery. In contrast, the relaxin concentration in serum was not elevated after SAH. The relaxin concentration in serum is affected by systemic conditions such as pregnancy, heart failure, and cancer [3, 29, 30]. The relaxin concentration in CSF may be affected by intracranial conditions after SAH. Arterial-derived relaxin is produced locally and acts in an autocrine/paracrine manner [8, 13]. Therefore, the elevated relaxin concentration in CSF indicates that relaxin is produced in the basilar arterial wall and secreted into the CSF. Furthermore, the time course of the relaxin concentration in CSF was correlated with the progression of cerebral vasospasm after SAH. Thus, the relaxin concentration in CSF may be a potential biomarker for detecting the presence of cerebral vasospasm after SAH.

In conclusion, we demonstrated that* RLN1* mRNA was gradually upregulated after SAH, whereas* RXFP1* mRNA and protein were downregulated just after SAH. The relaxin concentration in CSF was significantly elevated after SAH, and the time course was consistent with that of expression of* RLN1* mRNA in the rabbit basilar artery. These findings suggest that expression and signaling of relaxin and RXFP1 participate in the development of cerebral vasospasm after SAH. Moreover, downregulation of RXFP1 may cause a functional reduction in relaxin activity in cerebral arteries during vasospasm. Further studies are needed to clarify the role of relaxin in the development of cerebral vasospasm.

## Figures and Tables

**Table 1:** The top three most highly upregulated genes in the rabbit basilar artery after subarachnoid hemorrhage identified with microarray analysis.

	Day 3		Day 5		Day 7	
	Gene	log_2_⁡FC	Gene	log_2_⁡FC	Gene	log_2_⁡FC
1	SAA	6.02	RLN1	4.73	RLN1	4.82
2	SAA3P	5.88	SAA3P	3.50	SAA3P	3.93
3	HP	5.78	HP	3.40	HP	3.47

SAA: serum amyloid A; SAA3P: serum amyloid A3 protein; HP: haptoglobin; RLN1: relaxin-1; log_2_⁡FC: log_2_⁡ fold change.
